# NCL1, A Highly Selective Lysine-Specific Demethylase 1 Inhibitor, Suppresses Castration-Resistant Prostate Cancer Growth via Regulation of Apoptosis and Autophagy

**DOI:** 10.3390/jcm8040442

**Published:** 2019-03-31

**Authors:** Toshiki Etani, Taku Naiki, Aya Naiki-Ito, Takayoshi Suzuki, Keitaro Iida, Satoshi Nozaki, Hiroyuki Kato, Yuko Nagayasu, Shugo Suzuki, Noriyasu Kawai, Takahiro Yasui, Satoru Takahashi

**Affiliations:** 1 Department of Nephro-Urology, Nagoya City University, Graduate School of Medical Sciences, Nagoya 467-8601, Japan; uroetani@med.nagoya-cu.ac.jp (T.E.); ikeitarou1009@gmail.com (K.I.); snozaki@med.nagoya-cu.ac.jp (S.N.); n-kawai@med.nagoya-cu.ac.jp (N.K.); yasui@med.nagoya-cu.ac.jp (T.Y.); 2 Department of Experimental Pathology and Tumor Biology, Nagoya City University, Graduate School of Medical Sciences, Nagoya 467-8601, Japan; ayaito@med.nagoya-cu.ac.jp (A.N.-I.); h.kato@med.nagoya-cu.ac.jp (H.K.); naga-p@dk.pdx.ne.jp (Y.N.); shugo@med.nagoya-cu.ac.jp (S.S.); sattak@med.nagoya-cu.ac.jp (S.T.); 3 Department of Chemistry, Kyoto Prefectural University of Medicine, Graduate School of Medical Science, Kyoto 606-0823, Japan; suzukit@koto.kpu-m.ac.jp; 4 CREST, Japan Science and Technology Agency (JST), Kawaguchi 332-0015, Japan

**Keywords:** LSD1, epigenetics, castration-resistant prostate cancer, autophagy

## Abstract

Recent studies have shown that epigenetic alterations lead to oncogenic activation, thus indicating that these are therapeutic targets. Herein, we analyzed the efficacy and therapeutic potential of our developed histone lysine demethylase 1 (LSD1) inhibitor, NCL1, in castration-resistant prostate cancer (CRPC). The CRPC cell lines 22Rv1, PC3, and PCai1CS were treated with NCL1, and LSD1 expression and cell viability were assessed. The epigenetic effects and mechanisms of NCL1 were also evaluated. CRPC cells showed strong LSD1 expression, and cell viability was decreased by NCL1 in a dose-dependent manner. Chromatin immunoprecipitation analysis indicated that NCL1 induced histone H3 lysine 9 dimethylation accumulation at promoters of P21. As shown by Western blot and flow cytometry analyses, NCL1 also dose-dependently induced caspase-dependent apoptosis. The stimulation of autophagy was observed in NCL1-treated 22Rv1 cells by transmission electron microscopy and LysoTracker analysis. Furthermore, WST-8 assay revealed that the anti-tumor effect of NCL1 was reinforced when autophagy was inhibited by chloroquine in 22Rv1 cells. Combination index analysis revealed that a concurrent use of these drugs had a synergistic effect. In ex vivo analysis, castrated nude mice were injected subcutaneously with PCai1 cells and intraperitoneally with NCL1. Tumor volume was found to be reduced with no adverse effects in NCL1-treated mice compared with controls. Finally, immunohistochemical analysis using consecutive human specimens in pre- and post-androgen deprivation therapy demonstrated that LSD1 expression levels in CRPC, including neuroendocrine differentiation cases, were very high, and identical to levels observed in previously examined prostate biopsy specimens. NCL1 effectively suppressed prostate cancer growth in vitro and ex vivo without adverse events via the regulation of apoptosis and autophagy, suggesting that NCL1 is a potential therapeutic agent for CRPC.

## 1. Introduction

Prostate cancer is one of the most frequently diagnosed cancers in Western countries. In advanced prostate cancer, androgen deprivation therapy (ADT) has remained a first-line therapy for decades [1]. After showing an initial response, most patients develop progressive disease, referred to as castration-resistant prostate cancer (CRPC). Intriguingly, CRPC is not androgen independent and several new drugs designed to further suppress the androgen receptor (AR) pathway have led to improved survival, including abiraterone acetate and enzalutamide [2,3,4,5]. The human AR gene encodes for a protein with an atomic mass of 110 kDa that consists of an N-terminal domain, a DNA-binding domain, and a ligand-binding domain. AR controls the growth of the prostate gland, and much evidence from preclinical and clinical studies has shown that multiple androgen/AR signaling pathways implicated throughout the various stages of prostate cancer [6]. In addition, recent reports have described the potential therapeutic implications of estrogen and related receptors in prostate cancer [7,8]. However, not all patients respond equally to these newer AR-targeting drugs. Approximately 20–40% of patients with CRPC have a poor clinical response to such agents, and nearly all patients who initially respond acquire secondary resistance. Prospective trials are ongoing to develop the best biomarker strategy for identifying patients resistant to these drugs.

Prostate cancer progresses in a multistep process in response to changes in genetic mechanisms. However, in addition to genetic mutations, epigenetic alterations have also been identified as activating oncogenes and causing a loss of function of tumor suppressor genes [9,10]. Methylation is a form of post-translational covalent modification of histones that epigenetically regulates specific gene expression patterns. Lysine-specific demethylase 1 (LSD1), a member of the flavin adenine dinucleotide dependent enzyme family, behaves like a histone demethylase. LSD1 acts by removing one or two methyl (but not three) groups from lysine residues 4 or 9 in histone H3 (H3K4 and H3K9, respectively) [11].

Growing evidence indicates that LSD1 is critical for human tumorigenesis, and its expression is increased in several malignancies, including prostate, breast, lung, ovarian, and colon cancers [12,13,14,15,16]. Therefore, the inhibition of LSD1 holds promise as a novel anticancer strategy. We have previously developed a novel and selective LSD1 inhibitor called NCL1 (*N*-[(1S)-3-[3-(trans-2-aminocyclopropyl)phenoxy]-1-(benzylcarbamoyl)propyl] benzamide) after using a combination of in vitro screening and protein structure similarity clustering [17]. In addition, we have reported that NCL1 impairs LSD1 demethylase activity and inhibits cell proliferation in castration-naïve prostate cancer [16].

In this study, we examined the LSD1 status in CRPC cell lines and human specimens including aggressive neuroendocrine differentiated (NED) phenotypes. In addition, we tested the therapeutic significance of NCL1 in CRPC cells in vitro and in an ex vivo subcutaneous model. Furthermore, we investigated the pharmacological mechanism of NCL1 using chromatin immunoprecipitation (ChIP), flow cytometry, and Western blot analyses. To our knowledge, we are the first laboratory to describe the inhibition of LSD1-induced cell death in CRPC through the regulation of autophagy by NCL1.

## 2. Results

### 2.1. LSD1 Is Highly Expressed in CRPC

**Figure 1:**
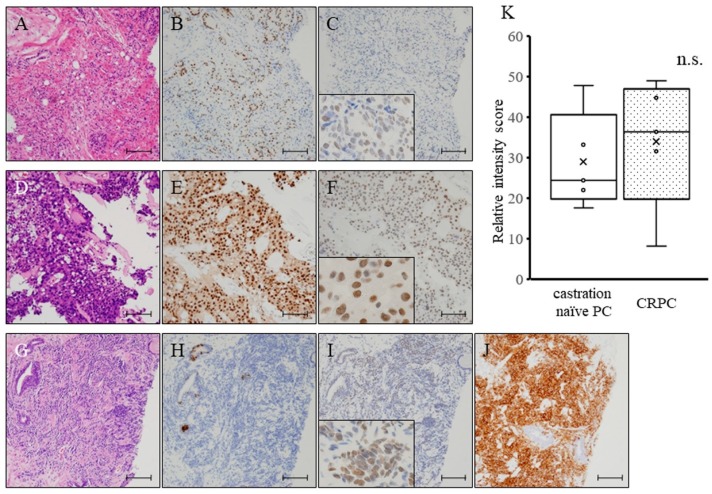
(**A**–**F**) Hematoxylin and eosin (HE) staining (**A**), and immunohistochemistry for Nkx3.1, a sensitive specific marker of differentiated adenocarcinoma originating from the prostate (**B**), and histone lysine demethylase 1 (LSD1) (**C**) of castration-naïve prostate cancer (castration-naïve PC) specimens obtained by prostate biopsy for an initial diagnosis in patients. HE staining (**D**), and immunohistochemistry for Nkx3.1 (**E**), and LSD1 (**F**) of castration-resistant prostate cancer (CRPC) specimens obtained by prostate biopsy after the acquisition of castration resistance and treatment with androgen deprivation therapy. Nuclei were counterstained with hematoxylin. Scale bar is 50 μm. (**G**–**J**) HE staining (**G**), and immunohistochemistry for Nkx3.1 (**H**), LSD1 (**I**), and synaptophysin (**J**) in prostate biopsy specimens obtained after the acquisition of castration resistance and neuroendocrine differentiation after treatment with androgen deprivation therapy. Scale bar is 50 μm. (**K**) A graphical comparison of intensity levels of LSD1 expression between castration-naïve PC and CRPC biopsy samples. n.s.: not significant.

To determine the status of LSD1 in human prostate cancer, the expression of LSD1 and Nkx3.1, a sensitive specific marker of differentiated adenocarcinoma originating from the prostate [18], was examined in human prostate biopsy specimens by immunohistochemistry and the staining intensity scored. We found that LSD1 expression levels in CRPC were very high, as previously found in prostate biopsy specimens obtained from patients (Figure 1A–F,K). Interestingly, neuroendocrine-differentiated tumors after androgen deprivation therapy, which had no expression of Nkx3.1, had high levels of LSD1 expression (Figure 1G–J). 

### 2.2. LSD1 Expression in Prostate Cancer Cell Lines and Suppression of Prostate Cancer Cell Proliferation by NCL1

**Figure 2:**
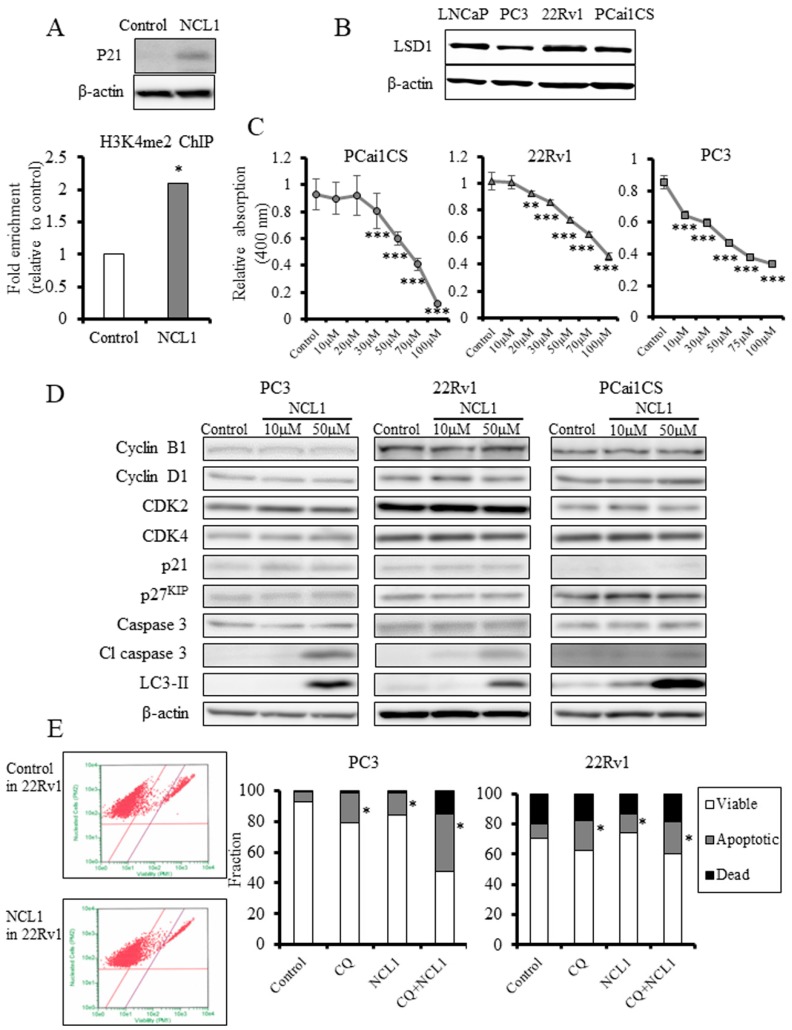
(**A**) Chromatin immunoprecipitation (ChIP) analysis in 22Rv1 cells using a histone H3 lysine 4 dimethylation (H3K4me2) antibody showed that NCL1 induced the attenuation of demethylation of H3K4me2 in the promoter regions of P21. Western blot analysis of P21 in 22Rv1 cells is shown. The protein expression of P21 was increased, reflecting the results of the ChIP analysis. β-actin was used as an internal loading control. (**B**) Western blot analysis of PCai1CS, 22Rv1, and PC3 cells for LSD1. All castration-resistant prostate cancer (CRPC) cell lines expressed LSD1. β-actin was used as an internal loading control. (**C**) PCai1CS and 22Rv1 cells were treated with vehicle (control) or NCL1, and subjected to WST-8 assay to measure cell proliferation. NCL1 treatment reduced the cell viability of the two CRPC cell lines in a dose-dependent manner. (**D**) Western blot analyses 48 h after NCL1 treatment of 22Rv1, PCai1CS, and PC3 cells. The cell cycle-related protein expression of cyclin B1, cyclin D1, CDK2, CDK4, and p27^KIP^ were unchanged. Treatment with NCL1 resulted in a marked elevation in cleaved caspase 3 without any change in caspase 3. In addition, protein expression of microtubule-associated protein light chain 3 (LC3)-II was elevated in NCL1-treated CRPC cells. β-actin was used as an internal loading control. (**E**) Guava^®^ apoptosis analysis of PC3 and 22Rv1 cells. NCL1, the autophagy inhibitor chloroquine (CQ), and a combination of these drugs induced apoptosis in CRPC cells. Mean ± standard deviation (SD); * *p* < 0.05, ** *p* < 0.001, *** *p* < 0.0001.

First, to determine whether LSD1 inhibition influences specific gene methylation status, 22Rv1 cultured prostate cancer cells treated with NCL1 were subjected to chromatin immunoprecipitation (ChIP) assay. As a result, and consistent with our previous report, NCL1 specifically impaired the demethylation of histone H3 lysine 4 (H3K4me2) at the containing promoter lesion of P21 genes (Figure 2A), reflecting the increased level of fold enrichment compared with the IgG control in ChIP assay, and increased levels of P21 protein expression in Western blot analysis, compared to the control. Next, we examined the status of LSD1 in CRPC cell lines, and found by Western blot analysis that LSD1 protein was highly expressed (Figure 2B). The proliferation of prostate cancer cells was significantly decreased by NCL1 treatment in a dose-dependent manner in the cancer cell lines tested, as determined by cell proliferation assay (Figure 2C). These findings suggest that NCL1 attenuated CRPC cell proliferation by demethylating H3K4me2 via LSD1 inhibition.

### 2.3. NCL1 Inhibits CRPC Cell Growth by Apoptotic Mechanisms

To determine how NCL1 induced growth inhibition, proteins involved in the cell cycle and apoptosis were examined in NCL1-treated CRPC cell lines. Reflecting the inhibition of LSD1, the expression of P21 was enhanced (Figure 2A). Cleaved caspase 3 was markedly elevated after treatment with NCL1 but caspase 3 expression remained unchanged. However, examination of cell cycle-related proteins showed that cyclin B1, cyclin D1, cyclin-dependent kinase (CDK)2, CDK4, and p27^KIP^ expression were not changed by NCL1 treatment (Figure 2D). Therefore, analyses by Guava^®^ ViaCount assay were performed. As a result, we found that NCL1 treatment of PC3 and 22Rv1 cells led to a significant induction of apoptosis (Figure 2E). These results suggested that selective attenuation of LSD1 using NCL1 inhibits cell proliferation by caspase-dependent apoptosis.

### 2.4. NCL1 Potentially Regulates Autophagy to Induce Cell Death in 22Rv1 Cells

**Figure 3:**
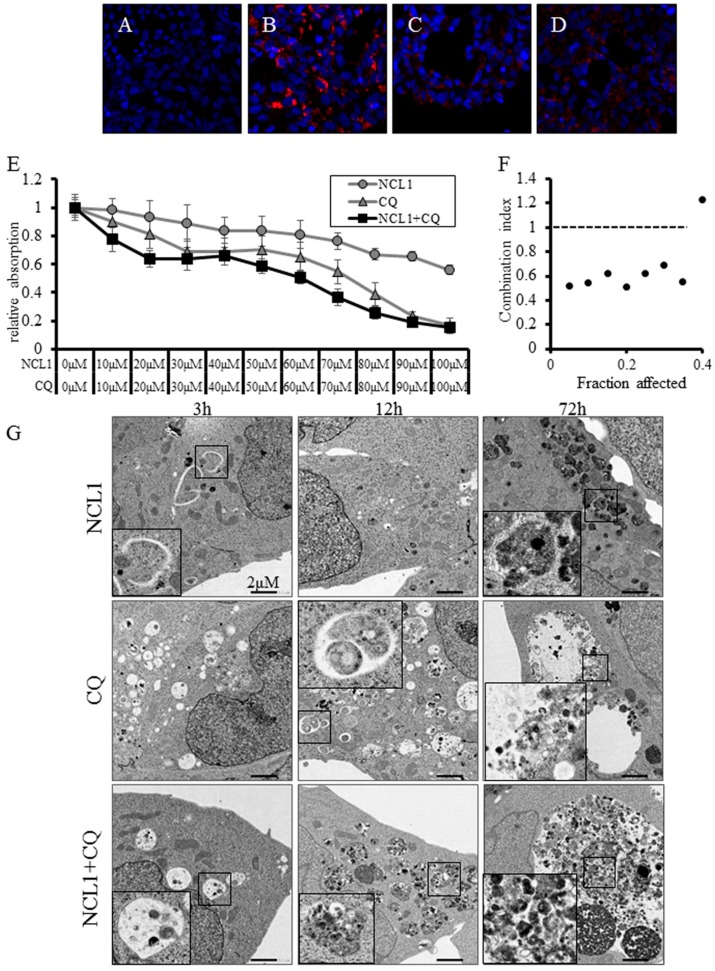
(**A**–**D**) Detection of the activation of lysosomes using LysoTracker analysis in 22Rv1 cells. Cells were treated with vehicle control (**A**), 50 μM NCL1 (**B**), 50 μM chloroquine (CQ) (**C**), or with 50 μM NCL1 and 50 μM CQ (**D**). Blue: nuclei, red: lysosomes. (**E**) 22Rv1 cells were treated with 50 μM NCL1 and/or 50 μM CQ. A WST-8 assay, in which the dye absorption rate positively correlated with cell viability, revealed that a combination of NCL1 and CQ decreased cell growth. (**F**) A combination index was calculated from the results of the WST-8 assay in Figure 3E. The combination of NCL1 and CQ showed a synergistic effect. (**G**) Cells were treated with 50 μM NCL1 for 3 h, 12 h, and 72 h. Three hours after NCL1 treatment, the formation of autophagosomes was noted by transmission electron microscopy (TEM). The cytoplasm also showed increased numbers of structures (visible in the 72 h figure) from 24 h to 72 h. Scale bar is 20 μm.

The conversion of microtubule-associated protein light chain 3 (LC3)-I to LC3-II and the formation of LC3 puncta were used to determine whether NCL1 treatment induced autophagy in CRPC cells. We found that NCL1 induced an increase of LC3-II protein levels in 22Rv1, PCai1CS, and PC3 cells as determined by Western blotting (Figure 2D). Therefore, to confirm the contribution of NCL1 to autophagy, we then raised the pH of the lumen of lysosomes and/or autolysosomes to inhibit autophagic flux using chloroquine (CQ), thereby preventing autophagic degradation. Flow cytometry revealed that a combination of NCL1 and CQ increased apoptotic cell numbers (Figure 2E). In addition, it was revealed by LysoTracker analysis that NCL1 treatment led to a further accumulation of activated lysosomes (Figure 3B), and the addition of CQ caused an attenuation of the phenomenon (Figure 3D). By WST-8 cell counting assay, CQ alone was shown to have an effect on 22Rv1 cell viability, while CQ enhanced the inhibition of cell growth by NCL1 (Figure 3E). Furthermore, combination index analysis revealed that the force of combination was shown to be synergistic (Figure 3F). NCL1 treatment for 3 h led to the formation of autophagosomes, as shown in Figure 3G. The cytoplasm also showed an increase in structures (shown in the 72 h figure) from 24 h to 72 h; the results obtained with LysoTracker suggest that these structures were lysosomes. CQ treatment led to the inhibition of the degradation of structures incorporated into phagosomes. Using a combined treatment, these findings revealed colocalization (Figure 3G). These results suggest that NCL1 may induce CRPC cell death by regulating autophagy potential in addition to regulating an apoptotic anticancer pathway. 

### 2.5. Ex Vivo Regulation of Tumorigenesis by NCL1

**Figure 4:**
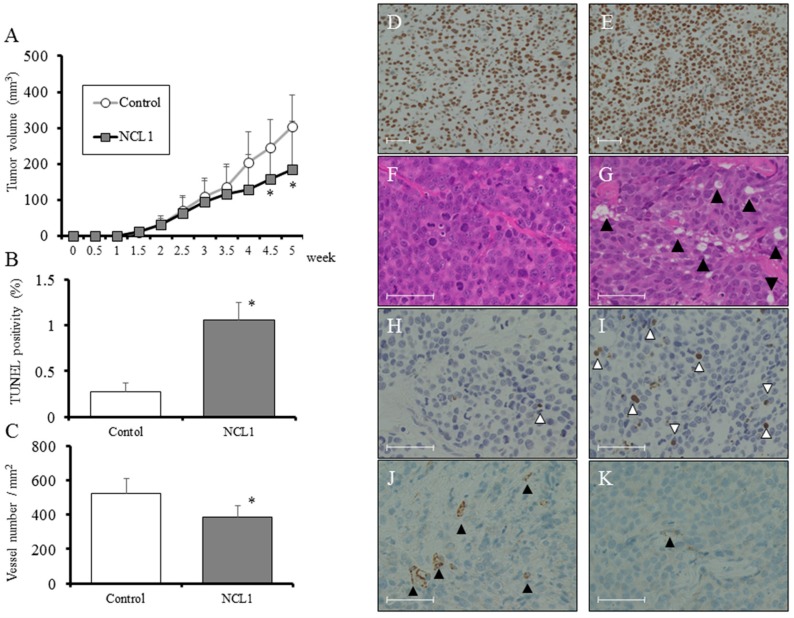
(**A**) Tumor growth was significantly inhibited in mice treated with 1.0 mg/kg NCL1 as compared to vehicle controls. (**B**) A terminal deoxy nucleotidyl transferase-mediated dUTP nick end labeling (TUNEL) assay was performed in NCL1-treated and control mice, and quantified as the mean TUNEL labeling percentage based on at least five randomly selected high-power microscope fields per individual. (**C**) Immunohistochemistry for CD31. Positivity was quantified as the mean number of vessels/mm^2^ based on at least five randomly selected high-power microscopic fields per individual. (**D**,**E**) Representative immunohistochemistry of LSD1 in a subcutaneous PCai1 tumor. Uncastrated tumor (**D**), and 1 week after castration (**E**). Scale bar is 50 μm. (**F**,**G**) Hematoxylin and eosin (HE) staining in subcutaneous tumors from vehicle control (**F**) and 1.0 mg/kg NCL1-treated (**G**) mice. Vacuolation (black arrowheads) was increased in the NCL1-treated group compared with controls. Scale bar is 50 μm. (**H**,**I**) TUNEL staining for apoptosis in subcutaneous tumors from vehicle control (**H**) and 1.0 mg/kg NCL1-treated (**I**) mice. White arrowheads indicate TUNEL-positive cells. Scale bar is 50 μm. (**J**,**K**) Representative immunohistochemical images of CD31 in subcutaneous tumors from control (**J**) and 1.0 mg/kg NCL1-treated (**K**) mice. Black arrowheads indicate CD31-positive cells. Scale bar is 50 μm. Mean ± standard deviation (SD); * *p* < 0.05.

We examined the expression level of LSD1 after castration in a PCai1 subcutaneous tumor model. We found a high level of LSD1 expression that remained unchanged 1 week after castration (Figure 4D,E), and continued at this level for 8 weeks. We next assessed the role of NCL1 in tumor progression ex vivo. After PCai1 cells were injected subcutaneously into castrated nude mice, animals were subsequently treated with vehicle control or 1.0 mg/kg of NCL1. The NCL1-treated group showed a significant inhibition of tumor size compared to vehicle controls (Figure 4A). The size of other organs and body weights were not affected by NCL1 treatment, and differences in the relative weights of organs and blood parameters between the two groups were not found (Table 1 and Table 2). Vacuolization was found to be increased in the groups treated with NCL1 compared with the vehicle control group (Figure 4F,G). Mechanisms involved in the inhibition of tumor growth by NCL1 in an animal model were examined using terminal deoxy nucleotidyl transferase-mediated dUTP nick end labeling (TUNEL) assays. Increased numbers of TUNEL-positive cells, and therefore apoptosis, were noted after treatment with NCL1 compared with vehicle (Figure 4B,H,I). In addition, we undertook immunohistochemical staining of CD31 to examine tumor vascularity. NCL1-treated tumors were found to have significantly decreased numbers of CD-31 positive blood vessels (Figure 4C,J,K). These results suggest that NCL1 regulates apoptosis to induce cell death and decrease tumor vascularity, both in vitro and ex vivo.

## 3. Discussion

In the present study, we examined whether CRPC proliferation is repressed by LSD1 and targeted this molecule using a specific inhibitor, NCL1. The inhibition of LSD1 by NCL1 led to an increase in H3K4me2 modifications in the promoter region (Figure 2A) and induced increases in P21 protein expression (Figure 2A). NCL1 significantly inhibited growth in vitro (Figure 2C) as well as tumor growth ex vivo at low concentration levels (Figure 4A). In addition, adverse events were not noted in the general condition of the mice and in their blood analyses (Table 1-2). These results demonstrated the safety and efficacy of NCL1 in CRPC, highlighting its potential as a new treatment for this disease. We are the first to demonstrate the therapeutic potential of NCL1 ex vivo using a CRPC animal model.

Currently, CRPC patients are treated by ADT, including AR- and non-AR-targeting drugs [19]. However, the selection of more appropriate treatment and the sequencing of these drugs is increasingly being investigated. Above all, NED phenotypes have been identified in about 50% of cases of CRPC, which express NED markers such as synaptophysin and chromogranin [20]. The presence of NED markers has been shown to indicate a poorer prognosis when treated with AR-targeting drugs, including enzalutamide [21,22]. In previous reports, including our previous article, the overexpression of LSD1 in prostate cancer was shown to be a predictive marker for aggressive tumor biology and tumor recurrence during therapy [16,23,24]. However, reports describing changes in LSD1 expression using consecutive pre- and post-ADT specimens do not exist. Our immunohistochemical analyses demonstrated that the overexpression of LSD1 in aggressive cancer was maintained in castration-resistant cancer cells (Figure 1A–F). In addition, although only in one case, the overexpression of LSD1 was maintained after changes to an NED tumor (Figure 1G–J). Furthermore, using a PCai1 ex vivo model, a high level of LSD1 expression was maintained from 1 week after castration (Figure 4D,E), and cell growth of castration-resistant PCai1 cells was effectively suppressed by NCL1 both in vitro and ex vivo. Therefore, NCL1 may have therapeutic potential for CRPC, including NED phenotypes, from an early phase after the acquisition of castration resistance. Further studies are needed to clearly test its in vivo potential in combination with ADT, as well as with AR-targeting drugs.

We observed high protein expression of LSD1 in CRPC cells (Figure 2B), and the inhibition of LSD1 activity using NCL1 to reduce cell proliferation in vitro (Figure 2C). In addition, the common mechanism of cell death induced both in vitro and ex vivo by NCL1 was revealed to be caspase-dependent apoptosis (Figure 2D,E and Figure 4A,B,H,I). Apoptosis is an active cell suicide process that maintains cellular homeostasis; however, cancer cells can override apoptotic cell death by upregulating anti-apoptotic machinery and/or downregulating pro-apoptotic programs [25,26]. It is generally accepted that autophagy can function as an adaptive response to maintain cell survival and growth [27,28]. A recent report reveals that the mammalian target of rapamycin (mTOR) inhibition protects cancer cells from apoptosis during nutrient limitation [29]. Several studies have established that autophagy is associated with drug resistance in prostate cancer cells to ADT and inhibitors of PI3K/Akt/mTOR signaling [30,31,32]. In a previous study, we reported that LSD1 inhibition stimulated autophagy in castration-naïve prostate cancer [16]. Therefore, to confirm this phenomenon in CRPC, we examined drug-induced autophagy in 22Rv1 cells by detecting LC3-II expression, as well as LysoTracker analysis, a non-specific autophagic marker, using TEM and Western blotting. We showed that NCL1 induced autophagy in 22Rv1, PC3, and PCai1CS cells in a concentration-dependent manner (Figure 2D). WST-8 assay revealed that the anti-tumor effect of NCL1 was reinforced when autophagy was inhibited by CQ in 22Rv1 cells. In addition, combination index analysis revealed that a combination of these drugs showed a synergistic effect (Figure 3F). These results suggest that the stimulation of autophagy in CRPC protects cells against anti-tumor agents through LSD1. Also, when treated in combination with drugs that regulate autophagy, NCL1 may be more effective in the suppression of CRPC growth. 

LSD1 plays a key role in many physiological functions. Previous studies have described how LSD1 inhibition reduces cell growth by affecting the expression of several genes involved in proliferation and the cell cycle [33,34,35,36,37]. Previous reports described that estrogen-induced demethylation of H3K9me2 by LSD1 caused reactive oxygen species-induced DNA damage and subsequently caused apoptosis via the regulation of phosphorylation with DNA damage repair enzymes in hormone-responsive cells [38,39]. In addition, a recent report described how a combination of LSD1 knockdown and cisplatin effectively suppressed the proliferation of PC3 cells, and that vascular endothelial growth factor, one of the most important promoters of angiogenesis, was downregulated by LSD1 siRNA treatment [40]. Our results suggest a similar mechanism. The inhibitory mechanisms of cancer growth by NCL1 appeared to be related not only to direct effects on cell proliferation but also to effects on angiogenesis, as shown by a reduction in CD 31-positive vessels ex vivo (Figure 4C,J,K). Recently, an abnormal mRNA splice variant of the androgen receptor, called AR-V7 [41,42], was shown to convey resistance to ADT [43,44]. In addition, in patients with metastatic CRPC, the presence of detectable AR-V7 transcripts in circulating tumor cells has been associated with a high positive predictive value for a non-response to AR-targeting agents, including enzalutamide, in several studies [45,46]. Prospective trials are ongoing to develop the best biomarker strategy for identifying treatment-resistant patients. Interestingly, in 2018, Regufe da Mota et al. [47] reported that LSD1 inhibition caused attenuation of the expression of not only wild-type AR, but also AR-V7. Further prospective trials using biomarkers to help select patients are warranted to evaluate the benefits of a strategic sequence of several drugs, including NCL1, for patients with CRPC.

## 4. Conclusions

In summary, NCL1 suppressed CRPC growth in vitro and ex vivo, showing strong efficacy without adverse events by regulating autophagy and apoptosis. Further, the strong expression of LSD1 was noted in human CRPC cells including those with NED phenotypes. These findings highlight how NCL1 may be considered a novel potential therapeutic agent for CRPC.

## 5. Materials and Methods

### 5.1. Human Castration-Naïve and Castration-Resistant Prostate Cancer Specimens

We obtained five castration-naïve prostate cancer specimens from five patients by needle biopsy. In addition, consecutive castration-resistant prostate cancer specimens after treatment were obtained by surgery or biopsy from the same patients from which previous biopsy specimens had been collected at Nagoya City University and affiliated hospitals between 2010 and 2016. All specimens were obtained after patients had provided written informed consent for the use of their tissues, according to an Institutional Review Board approved protocol with approval number 1168. All cases were evaluated by a panel of experienced pathologists.

### 5.2. Chemicals

NCL1 was synthesized as previously described [17].

### 5.3. Prostate Cancer Cell Lines

The human prostate cancer cell lines PC3 and 22Rv1 were obtained from the American Type Culture Collection (Rockville, MD, USA); these cells plus a castration-resistant rat prostate cancer cell line, PCai1, were cultured as previously described [46,47]. The cell lines used included PC3, which is human CRPC cell line without AR expression; 22Rv1, which is human CRPC cell line with AR expression; and PCai1CS, which is established from CRPC originating from a transgenic rat model as previously described [48,49]. They were cultured in media with 10% charcoal-stripped serum, and then treated with dimethyl sulfoxide (DMSO) as a vehicle that was equal in concentration to that used for 1–100 μM NCL1 for 72 h. Finally, its effect on cell proliferation determined. To assess the effects of autophagy on cell proliferation, PC3 and 22Rv1 cells were treated with 50 μM NCL1 and/or 50 μM chloroquine (CQ) for 24 h, an inhibitor of autophagy. All experiments were performed in triplicate.

### 5.4. Cell Proliferation Assay

A WST-8 Cell Counting Kit (Wako, Osaka, Japan) was used to assess the proliferation of cells grown in 96-well microplates. Prostate cancer cells were seeded in DMEM medium containing 10% fetal bovine serum (FBS) in 96-well plates (5 × 10^3^ cells/well), and a WST-8 assay was performed as previously described [16].

### 5.5. Analysis of the Cytotoxic Effect of NCL1 in Combination with Chloroquine

The effect of a drug combination was calculated according to the median effect principle. First, we constructed dose-response curves for the cytotoxic effects of NCL1 and chloroquine, both alone and in combination, in 22Rv1 cells using a WST-8 assay. The data were used to determine the ‘combination index’ (CI), using the equation: CI = (D)1/(Dx)1 + (D)2/(Dx)2, where (D)1 and (D)2 are the combinations doses that kill x% of cells, and (Dx)1 and (Dx)2 are the doses of each drug alone that kill x% of cells. If CI < 1, then synergism is indicated as previously described [50,51].

### 5.6. ChIP Assay

22Rv1 cells were incubated in the presence or absence of 50 μM NCL1 as indicated. Formaldehyde (1%) was used to cross-link cells, and the chromatin was collected and subjected to immunoprecipitation using an H3K4-me2 antibody (Cell Signaling Technology, Beverly, MA, USA). As a negative control, isotype-specific IgG was used. Extracted DNA was dissolved in TE buffer, and real-time PCR using specific primers (F-GGGGCGGTTGTATATCAGG, R-GGCTCCACAAGGAACTGACT) was used to confirm the methylation status of the promoter regions of P21 (CDKN1A).

### 5.7. Western Blot Analysis

Cells were lysed in SDS buffer, and 10 µL of protein lysate sample was dissolved in 12% polyacrylamide gels and transferred onto Hybond ECL membranes (GE Healthcare, Piscataway, NJ, USA). Antibodies against P21^WAF1^ (Cell Signaling), cyclin B1 (Santa Cruz Biotechnology, Santa Cruz, CA, USA), cyclin D1 (Santa Cruz), CDK2 (Santa Cruz), CDK4 (Santa Cruz), caspase 3 (Cell Signaling), cleaved caspase 3 (Cell Signaling), and LC3-II (Abcam, Cambridge, UK) were used to assess protein expression levels. A monoclonal anti-beta-actin antibody (Sigma, St. Louis, MO, USA) was used to evaluate beta actin expression as a protein loading control.

### 5.8. Flow Cytometry Analysis

PC3 and 22Rv1 cells (1 × 10^5^ per line) were treated with 50 μM NCL1 for 48 h, with or without 50 μM CQ, and cell suspensions were then prepared and stained with Guava^®^ ViaCount reagent and propidium iodide according to a Guava^®^ Assay protocol (Guava Technologies, Hayward, CA, USA). CytoSoft Software was used to analyze apoptosis and cell cycle phase distributions on a Guava^®^ PCA Instrument.

### 5.9. Lysosome Localization and Activity Using LysoTracker^®^ and LysoSensor™ Dyes

22Rv1 cells (3 × 10^4^) were seeded in 8-well chamber slides with RPMI medium and 5% FBS. Cells were incubated for 48 h and treated with 50 μM NCL1 or 50 μM CQ, or a combination of 50 μM NCL1 and 50 μM CQ. Control cells were treated with the same amount of solvent (DMSO and distilled water). We removed the medium from the dish after 48 h of treatment, and add the prewarmed (37 °C) probe-containing medium. The cells were then incubated for 60 minutes. Lastly, we replaced the loading solution with fresh medium and observed the cells using an IN Cell Analyzer 6000 (GE Healthcare, Chicago, IL, USA).

### 5.10. Transmission Electron Microscopy (TEM)

22Rv1 cells were seeded in 6-well plates (1 × 10^5^ cells/well) in DMEM containing 10% FBS. After an overnight incubation, cells were treated with or without 50 μM NCL1, and with or without 50 μM CQ for 3 or 72 h. Glutaraldehyde (2.5%) was used to pre-fix cells in 0.1 M phosphate buffer (pH 7.4) at 4 °C. Specimens were then post-fixed in 1% osmium tetroxide in 0.1 M phosphate buffer (pH 7.4) for 45 min. A graded series of ethanol was used to dehydrate specimens, which were subsequently embedded in epoxy resin. An Ultracut-S ultramicrotome (LEICA, Wetzlar, Germany) and a diamond knife were used to cut ultra-thin sections, which were then stained with 2% uranyl acetate in distilled water for 15 min and a lead staining solution for 5 min. A JEM-1011J (JEOL, Tokyo, Japan) electron microscope at 80 KV was used to observe sections.

### 5.11. Ex Vivo Studies Using a Subcutaneous Castration-Resistant PCai1 Model

Six-week-old male KSN/nu-nu nude mice from Nippon SLC (Hamamatsu, Japan) were maintained as previously described [48,49]. PCai1 cells cultured in T-75 flasks were grown to confluence, trypsinized, and counted. PCai1 cells (1 × 10^6^ in 100 μL serum-free DMEM) were subcutaneously injected into the dorsal side of each mouse under isoflurane anesthesia. After 1, 3, and 4 weeks, mice (*n* = 5) were castrated, while other mice (*n* = 5) were left uncastrated as negative controls. Five weeks after implantation, all mice were sacrificed, and the LSD1 expression of PCai1 tumors was analyzed. For the next experiment, all nude mice were castrated, and 1 × 10^6^ PCai1 cells resuspended in 100 μL serum-free DMEM were subcutaneously implanted as described above. Ten days later, an intraperitoneal injection of DMSO as a vehicle that was equal in concentration to that used for 1.0 mg/kg NCL1 (*n* = 10), or 1.0 mg/kg (*n* = 10) NCL1 was performed twice per week. Tumor size (determined by caliper measurement) and body weight were measured twice per week. Mice were sacrificed 5 weeks after the implantation of cells.

All animal experiments were performed according to protocols approved by the Institutional Animal Care and Use Committee of Nagoya City University Graduate School of Medical Sciences; the approval number was H24M-58.

### 5.12. Immunohistochemical Analysis

Deparaffinized tissue arrays were incubated with anti-LSD1 (1:200; Cell Signaling), anti-Nkx3.1 (1:400; Cell Signaling), or anti-synaptophysin (1:100; Cell Signaling). Deparaffinized animal tissues were incubated with anti-CD31 (1:100; Santa Cruz). Antibody binding was visualized by a conventional immunostaining method, as described previously [48,49], using an autoimmunostaining apparatus (HX System, Ventana, Tucson, AZ, USA).

LSD1 expression was evaluated using intensity scores for normal prostate glands and carcinoma cores from patients. For LSD1 immunoreactivity in nuclei, raw nuclear intensity data for tumor cells in prostate cancer cores and luminal cells in normal prostate glands were measured using a BZ-9000 multifunctional microscope and analysis software (Keyence Japan, Osaka, Japan). For each patient, evaluations were repeated five times and an average intensity score was calculated for each core.

### 5.13. TUNEL Assay 

A TUNEL assay using an in situ Apoptosis Detection Kit from Takara (Otsu, Japan) was used according to the manufacturer’s protocol to determine apoptotic cells in deparaffinized tissues. The relative ratio of TUNEL-positive cells was determined using five random microscopic fields for each group. 

### 5.14. Statistical Analysis

Student’s *t*, ANOVA, or Kruskal–Wallis tests were used to assess the association between variables. A value of *p* < 0.05 was considered statistically significant. 

## Figures and Tables

**Table 1:** Relative organ weights at the experiment’s termination in a PCai1 mouse tumor model. BW: body weight; R: right; L: left.

	No. of Mice	BW (g)	Liver (%)	R-Kidney (%)	L-Kidney (%)
Control	11	23.4 ± 1.3	5.14 ± 0.22	0.78 ± 0.02	0.80 ± 0.05
NCL1 1.0 mg/kg	10	23.5 ± 1.5	5.30 ± 0.26	0.81 ± 0.03	0.82 ± 0.04

**Table 2:** Blood results at the experiment’s termination in a PCai1mouse tumor model. AST: aspartate aminotransferase; ALT: alanine aminotransferase; ALP: alkaline phosphatase; T-Bil: total bilirubin; T-Chol: total cholesterol; Crea: creatinine; BUN: blood urea nitrogen; Na: sodium; K: potassium; Cl: chloride; Ca: calcium. Mean ± standard deviation (SD).

	Control	NCL1 1.0 mg/kg
AST	56.2 ± 10.3	55.9 ± 10.6
ALT	28.2 ± 6.3	27.1 ± 3.6
ALP	240.7 ± 20.6	250.2 ± 30.9
T-Bil	0.04 ± 0.01	0.04 ± 0.01
T-Chol	83.5 ± 8.3	85.1 ± 8.3
Crea	0.09 ± 0.01	0.10 ± 0.01
BUN	23.4 ± 3.2	23.5 ± 2.7
Na	146.3 ± 3.8	146.8 ± 2.7
K	6.7 ± 1.0	5.9 ± 0.7
Cl	110.4 ± 6.5	109.7 ± 5.3
Ca	8.5 ± 0.2	8.4 ± 0.3
